# Transmission of pancreatic adenocarcinoma by a single multiorgan donor to two kidney transplant recipients: A case report

**DOI:** 10.3389/fmed.2023.1142611

**Published:** 2023-03-14

**Authors:** Tanja Belčič Mikič, Gregor Mlinšek, Manca Oblak, Aljoša Kandus, Jadranka Buturović-Ponikvar, Simon Hawlina, Tomaž Milanez, Nika Kojc, Maja Frelih, Miha Arnol

**Affiliations:** ^1^Department of Nephrology, University Medical Centre Ljubljana, Ljubljana, Slovenia; ^2^Faculty of Medicine, University of Ljubljana, Ljubljana, Slovenia; ^3^Department of Urology, University Medical Centre Ljubljana, Ljubljana, Slovenia; ^4^Institute of Oncology Ljubljana, Ljubljana, Slovenia; ^5^Institute of Pathology, Faculty of Medicine, University of Ljubljana, Ljubljana, Slovenia

**Keywords:** kidney transplantation, cancer transmission, donor cancer, protocol biopsy, everolimus

## Abstract

We present two cases of transmission of a pancreatic adenocarcinoma from a single donor to two kidney transplant recipients. Autopsy of the donor revealed a pancreatic adenocarcinoma that had already spread locally to the regional lymph nodes and had not been detected at the time of organ procurement. Both recipients were carefully monitored, as neither consented to graft nephrectomy. In one patient, the tumor was discovered on surveillance biopsy of the graft approximately 14 months after transplantation, and in the second patient, ultrasound-guided aspiration needle biopsy of a growing formation in the lower pole of the graft revealed poorly differentiated metastatic adenocarcinoma. Both patients were successfully treated with graft nephrectomy and complete discontinuation of immunosuppression. None of the follow-up imaging showed persistent or recurrent malignancy, and both patients were candidates for re-transplantation. These exceptional cases of donor-derived pancreatic adenocarcinoma suggest that removal of the donor organ and restoration of immunity may lead to complete recovery.

## 1. Introduction

Despite careful donor selection, cancer transmission remains a rare but serious life-threatening complication of kidney transplantation. Here, we report one of the rarest cases of transmission of adenocarcinoma of the pancreas from a deceased single multiorgan donor to two kidney transplant recipients and its treatment after both patients refused removal of the kidney graft.

## 2. A report of two cases

### 2.1. The donor

The donor was a 46-year-old woman who died of spontaneous intracerebral hemorrhage. She had no known history of malignancy nor was there any evidence of malignancy listed in the Eurotransplant donor report. The other organs transplanted were the liver, heart, and lungs. Due to the patient’s history of diabetes, simultaneous transplantation of the kidney and pancreas was not considered.

### 2.2. Kidney recipients

The recipient of the right kidney was a 61-year-old man and the recipient of the left kidney was a 40-year-old man. Both kidney transplant recipients had been treated with dialysis for approximately 3 years (right kidney transplant recipient with hemodialysis and left kidney transplant recipient with peritoneal dialysis) and were successfully transplanted with a standard immunosuppression protocol (basiliximab induction, tacrolimus, mycophenolic acid, and methylprednisolone) in late November 2015. Methylprednisolone was discontinued in both patients after 1 week, and they were discharged with improving graft function (serum creatinine (SCr) of 161 μmol/l in the right kidney transplant recipient and 169 μmol/l in the left kidney transplant recipient).

**Figure 1:**
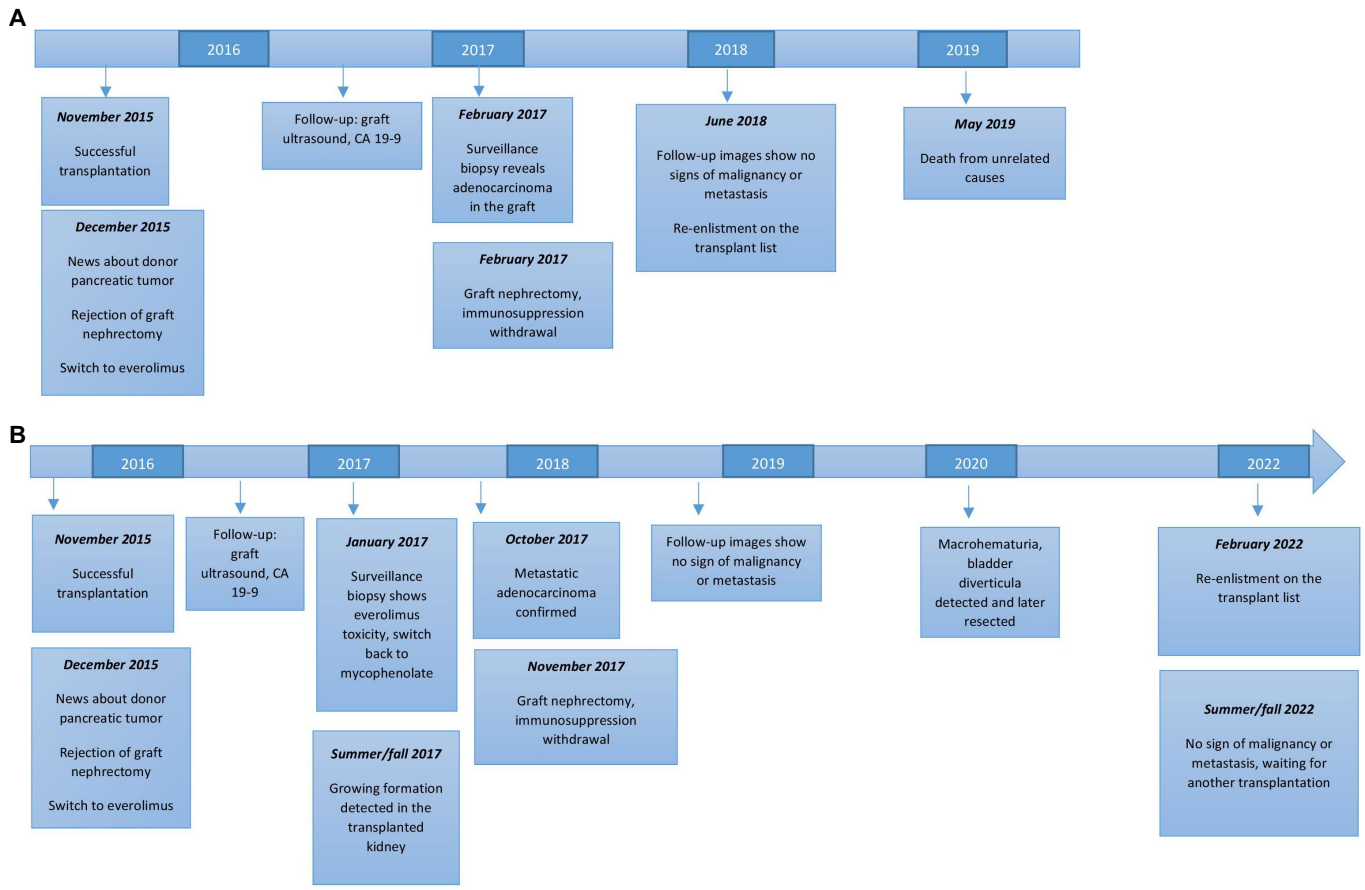
Timing of major events in both kidney transplant recipients. **(A)** Recipient of the right kidney. **(B)** Recipient of the left kidney.

On December 21, less than 1 month after transplantation, our center was informed by Eurotransplant that the donor had adenocarcinoma of the pancreas, which had already spread locally to the regional lymph nodes. The tumor was not macroscopically detectable at the time of donation and was only discovered at autopsy and later confirmed histologically. Because hematogenous spread of the pancreatic tumor appeared possible premortem, both recipients were offered immediate explantation of the graft. Unfortunately, neither patient consented to this procedure because of the tremendous burden of dialysis treatment. At this point, both patients were switched from mycophenolate to everolimus and dual immunosuppression with low-dose tacrolimus was continued. Both patients were followed-up closely. The timing of most important events for both recipients is presented in Figure 1.

**Figure 2:**
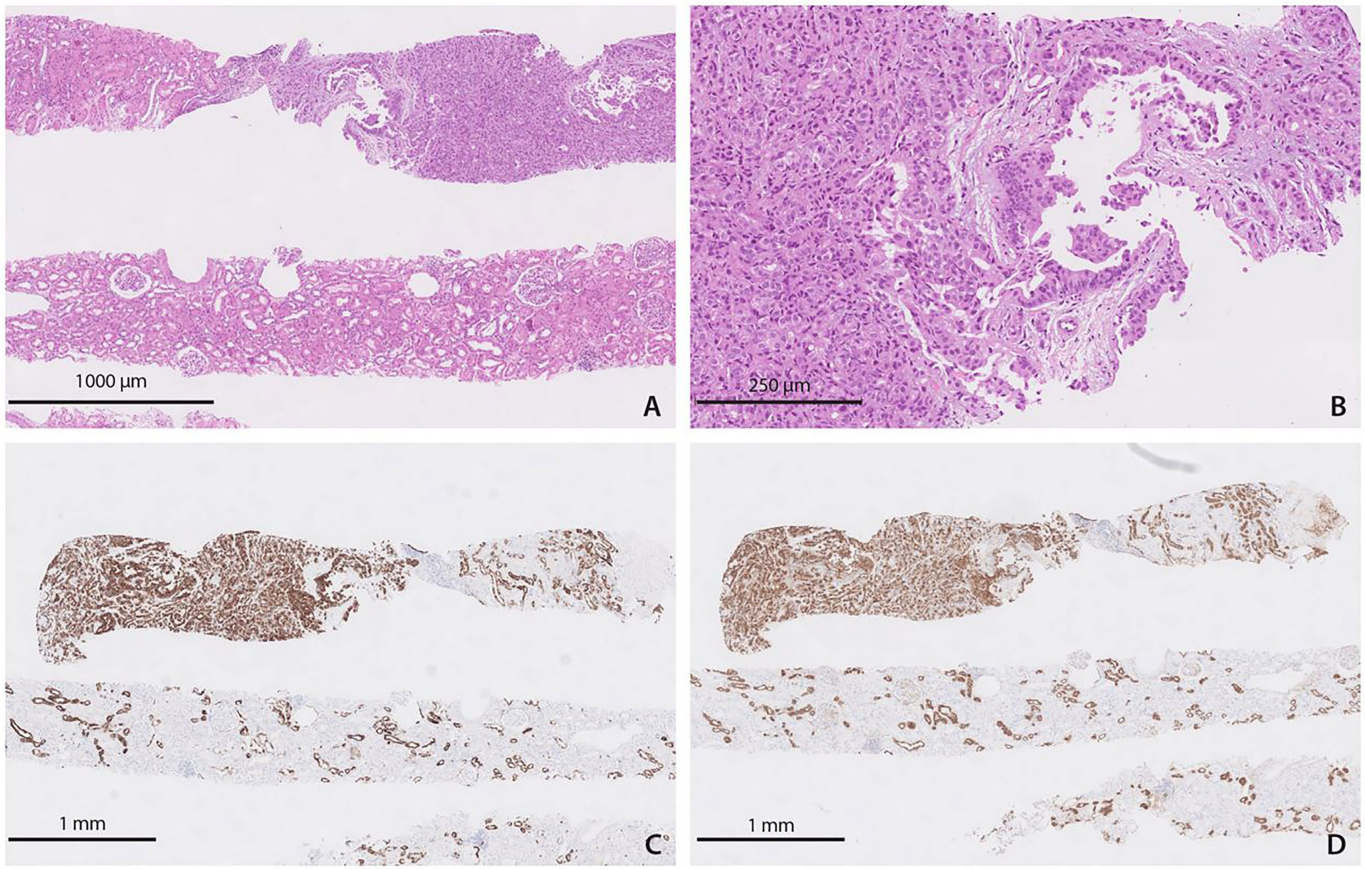
Histopathological findings in the recipient of the right kidney. **(A)** Adenocarcinoma in the surveillance biopsy of the graft [hematoxylin–eosin (H&E)]. **(B)** Adenocarcinoma in the surveillance biopsy of the graft – closer view (H&E). **(C)** Adenocarcinoma in the surveillance biopsy of the graft expressing cytokeratin 7 (immunohistochemical staining). **(D)** Adenocarcinoma in the surveillance biopsy of the graft expressing mucin-1 protein (immunohistochemical staining).

During the following year, the recipient of the right kidney was monitored with regular ultrasound examinations of the transplanted kidney and with the tumor marker carbohydrate antigen (CA) 19–9, and all test results were within the reference range. He declined monitoring with computed tomography (CT) because of the possibility of contrast-induced acute kidney injury. 12 months after transplantation, he was switched back to mycophenolate because of worsening lower extremity edema. A surveillance biopsy 14 months after transplantation revealed poorly differentiated adenocarcinoma in two of four tissue samples. Immunohistochemical findings (positive immunohistochemical staining for cytokeratin 19 and 7 and mucin-1 protein) suggested a pancreatic origin of the tumor (Figure 2). A sample of tumor tissue from the transplanted kidney was analyzed based on the detection of selected genetic markers on the X and Y chromosomes by the QF-PCR (Quantitative Fluorescence Polymerase Chain Reaction) method using the Aneufast Multiplex QF-PCR kit manufactured by molGENTIX (Spain), and the analysis confirmed the presence of a typical female profile with a small percentage of male profile (approximately 10%). Based on the combination of the timing of tumor development, the donor’s clinical history, histopathological/immunohistochemical profiles, and chromosomal characterization, a final diagnosis of metastatic (pancreatic) adenocarcinoma was made. At this time, the patient consented to graft nephrectomy. Before surgery, CT revealed multiple hypo-vascular lesions in the transplanted kidney suggestive of metastases. Immediately after graft nephrectomy, immunosuppressive therapy was discontinued. Thirteen days later, fluorodeoxyglucose positron emission tomography (FDG-PET) and contrast-enhanced magnetic resonance imaging (MRI) of the head showed no evidence of tumor metastasis. In June 2018, 30 months after transplantation, the patient was re-enlisted on the Eurotransplant waiting list, as none of the follow-up examinations (CT of the abdomen and thorax, FDG-PET and MRI of the head) showed signs of malignancy or metastases. Unfortunately, in May 2019, three and a half years after transplantation, the patient died suddenly and for unknown reasons, presumably of sudden cardiac death due to cardiac arrhythmia after a cherry meal. No autopsy was performed.

**Figure 3:**
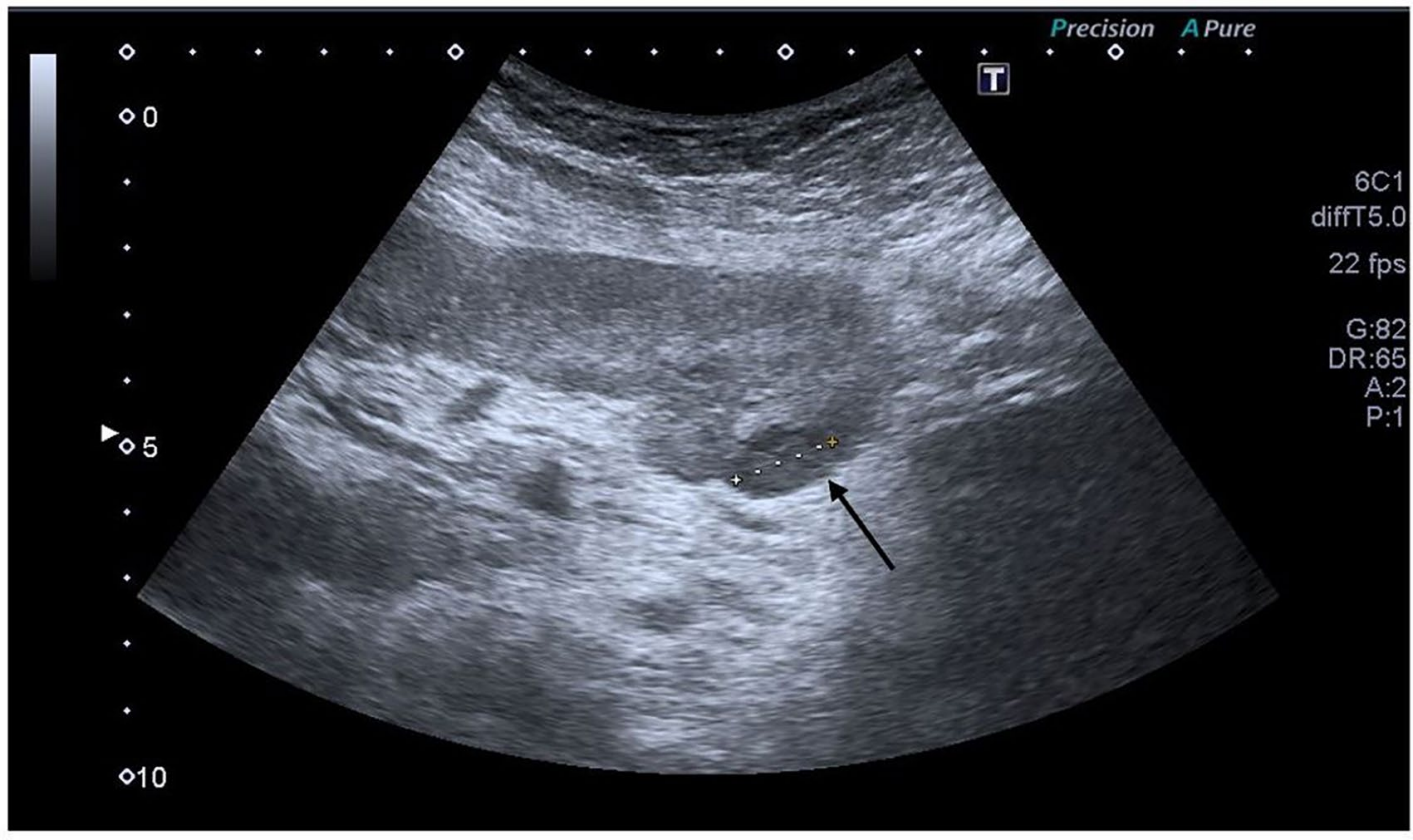
Ultrasound of the growing formation (arrow) in the kidney graft seen in the left kidney transplant recipient. An aspiration needle biopsy showed poorly differentiated cells of metastatic adenocarcinoma.

In the left kidney recipient, baseline contrast CT scans of the thorax and abdomen revealed no evidence of tumor. CA 19–9 was within the reference range. Follow-up abdominal ultrasound and CA 19–9 testing in the first year after transplantation were within the reference range. Approximately 12 months after transplantation, a graft biopsy was performed per protocol and indication (due to increasing proteinuria of more than 2 grams per day), which showed evidence of everolimus toxicity, leading to discontinuation of everolimus and reintroduction of mycophenolate. In February 2017, 14 months after transplantation, follow-up imaging diagnostics were performed, including CT of the abdomen and thorax, an MRI of the head, and an FDG-PET. The latter showed increased uptake of radiotracer in the anterior abdominal wall, at the site where a peritoneal dialysis catheter had previously been inserted; an aspiration needle biopsy was negative for tumor cells. The remaining examination findings were unremarkable. In summer and fall 2017, serial ultrasound examinations of the graft showed growing complex formation in the lower pole of the kidney (Figure 3). In October 2017, an aspiration needle biopsy revealed poorly differentiated cells from a metastatic adenocarcinoma. In November 2017, 2 years after transplantation, the graft was explanted, and immunosuppression was discontinued. The patient started hemodialysis.

**Figure 4:**
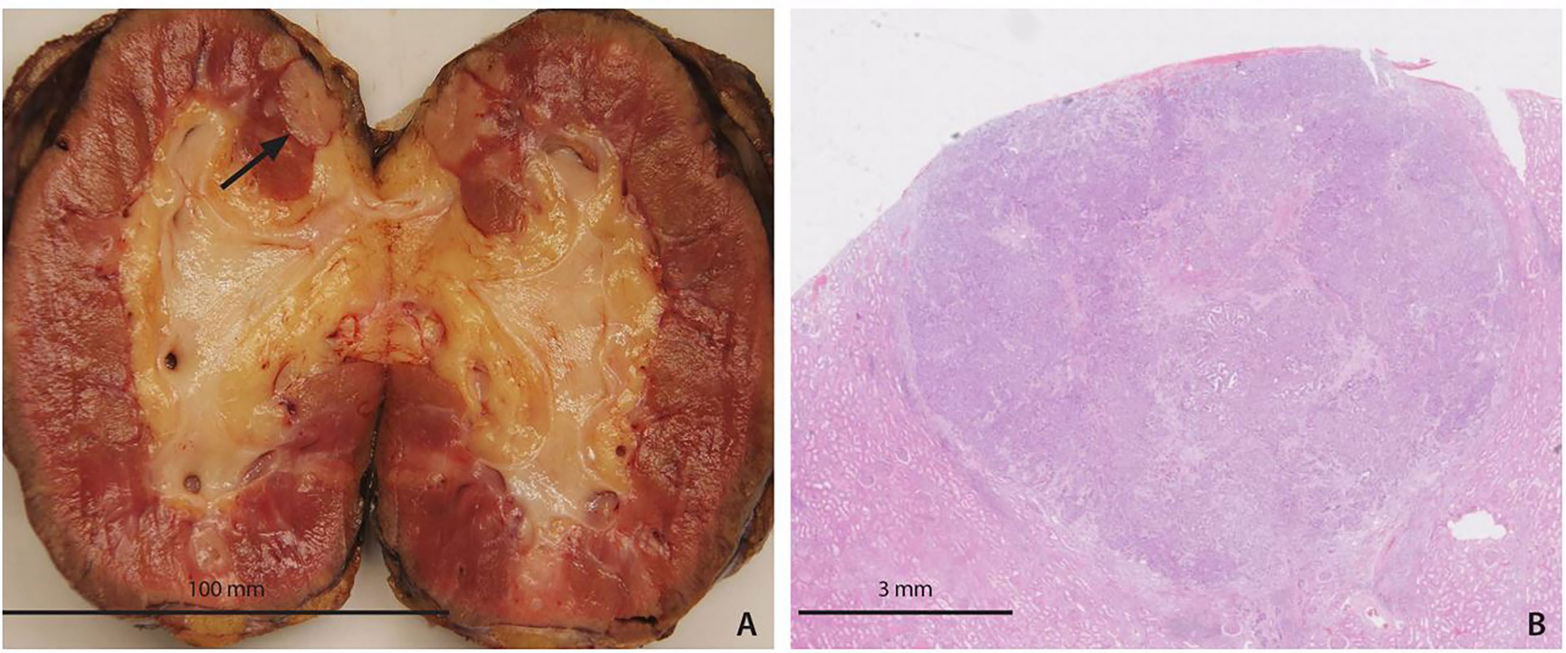
Pathological findings in the recipient of the left kidney. **(A)** Macroscopic view of the adenocarcinoma (arrow). **(B)** Adenocarcinoma by light microscopy (H&E).

Examination of the explanted kidney revealed an adenocarcinoma with identical immunohistochemical features as in the first recipient, suggesting a pancreatic origin of the tumor (Figure 4). Further imaging follow-up (CT of the thorax and abdomen and FDG-PET) 1 month after explantation and in the following years revealed no evidence of tumor or its metastatic spread. In June 2020, he presented with macrohematuria. Cystoscopy revealed bladder diverticula which were later robotically removed, and he was successfully placed back on the Eurotransplant waiting list. At his last follow-up in the fall of 2022 and almost 5 years after graft removal he was in good condition and awaiting another transplant.

### 2.3. Other organ recipients

At the time of discovery of pancreatic adenocarcinoma, all other organ recipients were in good health. However, except for the heart transplant recipient, their long-term survival was poor. The liver transplant recipient was immediately retransplanted but later died of metastatic pancreatic adenocarcinoma. The lung transplant recipient was also retransplanted and died 18 months after retransplantation from chronic rejection and multiorgan failure. Data on the other organ recipients were provided by Eurotransplant because they were treated at different European institutions.

## 3. Discussion

The cases we present are among the rare reports of pancreatic tumor transmission in organ transplantation that were successfully treated without recurrence of the tumor in any kidney recipients. Our patients were two adult men who refused removal of the kidney graft after autopsy of the donor revealed adenocarcinoma of the pancreas. Both were monitored closely, which allowed early detection of tumor transmission. Subsequent removal of the kidney graft and discontinuation of immunosuppression led to complete recovery in both recipients.

Transplantation of organs from donors with pancreatic cancer is considered an unacceptable risk because of its aggressive behavior and high risk of spread to other organs. Our donor was a 46-year-old woman with two known risk factors for pancreatic cancer, diabetes mellitus and cigarette smoking (1). Because of the donor’s history of diabetes, the pancreas was not transplanted. However, according to the Guide to the Quality and Safety of Organs for Transplantation (2), all organs in the thoracic and abdominal cavities, even if not considered for donation, should be fully investigated. This should allow detection of hidden tumor tissue or pathologic lymphadenopathy and avoid unwanted tumor transmission.

After refusal of explantation, both recipients were switched from mycophenolate to everolimus. It has been previously confirmed that everolimus significantly reduces the risk of *de novo* malignancy in kidney graft recipients even in patients receiving combination therapy with cyclosporine/tacrolimus (3). Furthermore, this mammalian target of rapamycin inhibitor may even have a role in the treatment of pancreatic adenocarcinoma, but this has only been suggested in preclinical trials and has not yet been clinically confirmed (4). Kidney graft recipients can be safely switched to everolimus after transplantation (5). However, several patients experience adverse effects that limit the use of everolimus (5), as was the case in our two recipients, one of whom developed nephrotoxicity with podocyte and tubular epithelial cell damage, which has been reported previously (6).

Data on the risk of pancreatic cancer transmission to the recipient remain sparse. Pancreatic cancer transmission has been reported to the Organ Procurement and Transplant Network (OPTN) as a possible donor-related malignancy in three recipients (7). Pancreatic tumor transmission has also been described in patients with combined pancreas-kidney transplantation (8, 9) and liver transplantation (10). In addition, in 2003, Gerstenkorn et al. described the case of a 56-year-old man who received a kidney from a patient with lymphangitis carcinomatosa of the lung, which was most likely of pancreatic origin. The functioning kidney was left in place, and the patient died 15 months after transplantation because of tumor spread to the pulmonary system (11). In contrast, in the cases we present, immunosuppression was completely discontinued at the time of confirmed tumor transmission and immediate graft nephrectomy. Graft nephrectomy and discontinuation of immunosuppression are the most common and successful forms of treatment in the unfortunate event of cancer transmission in kidney transplantation (12, 13). Successful treatment of donor-derived metastatic pancreatic tumors has already been described in an algorithm proposed for patients with metastatic disease after kidney/pancreas transplantation (9). The basis of treatment appears to be cessation of immunosuppression, which allows recovery of the recipient’s immune system that eliminates the donor tumor cells, and must inevitably be followed by nephrectomy of the graft. Oncologic therapy appears to be adjuvant (9, 12). In addition to treatment, early detection of the transmitted tumor is critical. In most transplant recipients, the transmitted cancer was diagnosed within the first 2 years after transplantation (12). This underscores that patients with potentially transmitted cancer should be closely monitored during the first 24 months after transplantation. In our recipients, the transmitted cancer was diagnosed 14 and 23 months after transplantation, respectively. Both recipients were carefully monitored with regular graft ultrasound examinations and cancer antigen 19–9 surveillance, as they were reluctant to undergo contrast-enhanced CT. However, in one patient, the tumor was not detected until the surveillance biopsy and had not previously been seen on imaging. This demonstrates the importance of the surveillance biopsy to detect not only subclinical rejection (14) but also possible microscopic tumor transmission. In our case, the results of the surveillance biopsy allowed us to detect the transmitted adenocarcinoma early and to respond in time. However, this was a rare and fortunate event, and in general, surveillance biopsy would be unlikely to detect tumor cells without a prior lesion.

In conclusion, our two cases show that even the unfortunate event of transmission of adenocarcinoma of the pancreas in kidney transplant recipients can be successfully treated without recurrence of the tumor. In a kidney graft lesion where tumor transmission is suspected, an ultrasound-guided biopsy is recommended for pathohistological evaluation. If preventive graft nephrectomy is not an option, immunosuppressive therapy should be adjusted and patients should be monitored radiologically on a regular basis, at least by periodic ultrasonography of the graft. Patients who have had a tumor transferred with the kidney graft in the past can be successfully placed on the waiting list for retransplantation.

## Data availability statement

The original contributions presented in the study are included in the article/supplementary material, further inquiries can be directed to the corresponding author.

## Author contributions

TBM: writing. GM, MO, AK, and MA: conceptualization, supervision, and writing. JB-P: supervision. NK and MF: pathology report, writing, and pathology supervision. SH: surgical supervision. TM: oncology supervision. All authors contributed to the article and approved the submitted version.

## Conflict of interest

The authors declare that the research was conducted in the absence of any commercial or financial relationships that could be construed as a potential conflict of interest.

## Publisher’s note

All claims expressed in this article are solely those of the authors and do not necessarily represent those of their affiliated organizations, or those of the publisher, the editors and the reviewers. Any product that may be evaluated in this article, or claim that may be made by its manufacturer, is not guaranteed or endorsed by the publisher.
